# Genetic Classification of a Novel Genotype of the Genus *Acanthamoeba* Isolated from Tap Water in Mexico

**DOI:** 10.3390/tropicalmed11040093

**Published:** 2026-04-01

**Authors:** Paloma Camacho-Aguilar, Leobardo Daniel Gonzalez-Zuñiga, Jose Reyes Gonzalez-Galaviz, Fernando Lares-Villa, Luis Fernando Lares-Jiménez, Luis Fernando Lozano Aguirre Beltrán, Alejandro Otero-Ruiz, Libia Zulema Rodriguez-Anaya

**Affiliations:** 1 Departamento de Biotecnología y Ciencias Alimentarias, Instituto Tecnológico de Sonora, Ciudad Obregón 85000, Sonora, Mexico; paloma.camacho207086@potros.itson.edu.mx (P.C.-A.); leobardo.gonzalez121194@potros.itson.edu.mx (L.D.G.-Z.); 2 Secretaría de Ciencia, Humanidades, Tecnología e Innovación (SECIHTI), Instituto Tecnológico de Sonora, Ciudad Obregón 85000, Sonora, Mexico; jose.gonzalez@itson.edu.mx; 3 Departamento de Ciencias Agronómicas y Veterinarias, Instituto Tecnológico de Sonora, Ciudad Obregón 85000, Sonora, Mexico; flares@itson.edu.mx (F.L.-V.); luis.lares@itson.edu.mx (L.F.L.-J.); 4 Unidad de Análisis Bioinformáticos, Centro de Ciencias Genómicas, Universidad Nacional Autónoma de México, Cuernavaca 62210, Morelos, Mexico; llozano@ccg.unam.mx; 5 Programa de Estancias Posdoctorales por México, Departamento de Ciencias de la Salud, Universidad de Sonora, Ciudad Obregón 85010, Sonora, Mexico; alejandro.otero@unison.mx

**Keywords:** *Acanthamoeba*, genotyping, whole genome sequencing, phylogenetic, pathogens, potable water

## Abstract

*Acanthamoeba* is a free-living amoeba (FLA) that causes the majority of human infections. It is found predominantly in aquatic environments and is classified according to morphology or genotype (T1-T23). Research on this FLA aims to monitor its distribution, identify existing genotypes, assess its infectious potential, and identify factors that contribute to its recurrence. This study performed a molecular characterisation of *Acanthamoeba* strains isolated from tap water in Cajeme, Sonora, Mexico, to classify their genotypes. This was complemented by whole-genome sequencing and mapping of the 18S rRNA region in a divergent strain, LUDO1, to obtain higher-resolution data for more reliable assessment of its divergence from known genotypes. Genotypes T4, T5, T11, and T15 were identified in the *Acanthamoeba*-specific amplimer S1 (ASA.S1) region using the maximum-likelihood method. The inclusion of the 18S rRNA region from strain LUDO1 enabled its classification as a new genotype (T24), with a dissimilarity exceeding 5% compared with the 23 known genotypes. Additionally, culture analysis revealed notable variation in trophozoite size among strains that correlated with phylogenetic sub-branching. This analysis contributed to the epidemiological understanding of *Acanthamoeba*’s high resistance to treatments and infection systems and demonstrated a broadening of the phylogenetic distribution within the genus.

## 1. Introduction

*Acanthamoeba* is one of the principal clinically relevant free-living amoebae (FLAs), recognized as a pathogen in human ocular infections and as an opportunistic agent in systemic infections [1]. It is able to thrive in different environments, such as soil, air in dust particles, and especially aquatic environments, including surface waters, recreational waters, bottled water, tap water, or wastewater, with a greater affinity for warm environments [2]. This protozoan has a higher incidence of reported infections in humans compared to other FLAs. It causes granulomatous amoebic encephalitis (GAE) in individuals with compromised immune systems, affecting the central nervous system (CNS). Although considered sporadic, it has a lethality rate of over 90% due to the difficulty in diagnosis and lack of specific treatment [3]. This protozoan also causes *Acanthamoeba* keratitis (AK), a highly painful corneal infection that often leads to vision loss. Additionally, it has been infrequently associated with sinusitis and cutaneous lesions and has been identified as a vector for other pathogenic microorganisms [4].

Its high resistance to stress conditions allows it to have a wide distribution of species worldwide. It is classified phenotypically into three groups based on the form and size of its cysts or genotypically through sequencing of the small subunit of the 18S ribosomal RNA (18S rRNA), with 23 genotypes identified to date, including both pathogenic and non-pathogenic strains [3,5]. In contrast to morphological classification, genotyping offers an independent alternative unaffected by environmental factors that may modify cyst morphology; thereby, it overcomes a major limitation in species identification while providing stability and reproducibility [3,6,7]. Today, various sequencing tools facilitate the development of this type of analysis that relies on genetic sequences, significantly impacting global epidemiological data [8,9].

Epidemiology contributes as an alternative approach to preventing *Acanthamoeba* infections [1,10]. In this regard, recreational or cultural activities have been closely linked to the development of infections caused by this amoeba, as people are exposed to the entry of cysts or trophozoites through nasal, ocular, or cutaneous lesions, and reports in tap water worldwide highlight a significant risk of infection, especially for contact lens wearers [6]. In the southern region of Sonora, Mexico, pathogenic species have been isolated from various aquatic environments; however, their presence in potable water is unknown due to the lack of studies [11,12]. Therefore, the objective of this study was to molecularly characterize *Acanthamoeba* strains isolated from tap water in Cajeme, Sonora, Mexico, by integrating genetic and genomic sequencing data for genotypic classification.

## 2. Materials and Methods

### 2.1. Identification of Drinking Water Treatment Plants and Sampling of Tap Water

Tap water samples were collected from September 2021 to March 2022 during the fall and winter seasons from Cajeme, Sonora, Mexico.

**Figure 1:**
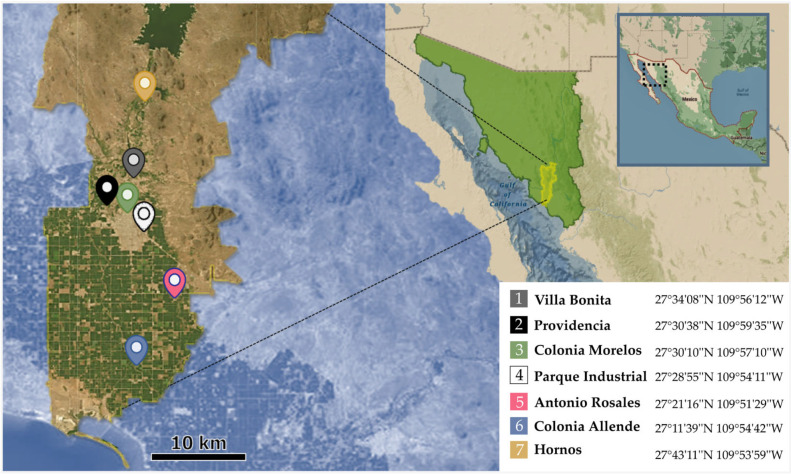
Drinking water treatment plants areas analysed in Cajeme, Sonora, Mexico (created with BioRender.com).

The Inventory of Municipal Purification and Wastewater Treatment Plants in Operation, December 2020, published by the Comisión Nacional de Agua (CONAGUA), was used to identify areas with potable water treatment plants [13]. Four facilities are located within the city’s urban areas, and three are in the surrounding rural areas (Figure 1).

For each water treatment plant, three nearby households were randomly selected, resulting in a total of 21 samples, to ensure that each sample originated exclusively from the treatment plant selected for analysis. At each household, 6 L of tap water was drawn into previously sterilized bottles [14]. Additionally, pH and chlorine levels were measured using a commercial kit (M601, Inter Water USA, San Francisco, CA, USA).

### 2.2. Sample Processing and Isolation of Acanthamoeba

Samples were processed by membrane filtration using cellulose membranes with a porosity of 1.2 μm (Merck Millipore, Burlington, VT, USA); each membrane was cut into two equal parts and placed (back to front) onto non-nutrient agar plates supplemented with *Escherichia coli* (NNE), resulting in a total of 6 plates per household (three plates were incubated at 37 °C and three at 42 °C) [14,15]. Continuous observation of NNE plates (every 24 h) was conducted over 72 h using an Axiovert 135 inverted microscope (ZEISS, Oberkochen, Germany) to identify and differentiate trophozoites of *Acanthamoeba*. Trophozoites were transferred to new NNE plates and incubated at 37 °C.

Isolates suspected of belonging to the genus *Acanthamoeba*, based on their morphological characteristics, were evaluated for proliferation in an axenic liquid medium to establish large-scale cultures and for subsequent molecular characterization. The axenic liquid medium used was Cerva’s medium (10.0 g of Bacto™ Casitone of Difco Laboratories Inc., Detroit, MI, USA) in a final volume of 500 mL of ddH_2_O, supplemented with 10% (*v*/*v*) fetal bovine serum, 200 IU/mL of penicillin, and 200 μg/mL of streptomycin as antibiotics), and cultures were incubated at room temperature (22–25 °C) [16].

### 2.3. DNA Extraction and Amplification of Acanthamoeba-Specific Amplimer S1 (ASA.S1) Region

DNA was extracted from *Acanthamoeba* trophozoites harvested from established in vitro cultures and collected by centrifugation at 4000× *g* for 10 min at room temperature, using the commercial kit Wizard SV Genomic DNA Purification System (Promega Corporation, Madison, WI, USA) following the manufacturer’s protocol for cell extraction. After DNA extraction, concentration and purity levels were measured using a NanoDrop 2000c equipment (Thermo Scientific, Wilmington, DE, USA).

The molecular evaluation was carried out by Polymerase Chain Reaction (PCR) using *Acanthamoeba*-specific primers, JDP1 (5′-GGCCCAGATCGTTTACCGTGAA-3′) and JDP2 (5′-TCTCACAAGCTGCTAGGGGAGTCA-3′), which amplify the diagnostic ASA.S1 region of the 18S rRNA gene, specific to the genus *Acanthamoeba* and enabling reliable genotypic discrimination [17]. For amplification, a reaction mix totalling 50 μL was prepared, containing 10 μL of Buffer, 4 μL of 25 mM MgCl_2_, 2 μL of each forward and reverse primer, 1 μL of dNTPs, 0.25 μL of 5x GoTaq Flexi polymerase (Promega Corporation, Madison, WI, USA), 10 μL of DNA, and the reaction was completed with 20.75 μL of nuclease-free water, and the thermocycler conditions were as follows: an initial incubation at 94 °C for 3 min, followed by 35 cycles of denaturation at 94 °C for 30 s, annealing at 55 °C for 30 s, extension at 72 °C for 30 s, and a final extension at 72 °C for 5 min [18].

### 2.4. Sequencing of the ASA.S1 Region and Initial Phylogenetic Analysis

Strains were selected for sequencing to include at least one representative strain per positive area, maximizing the geographic and genotypic diversity of the panel. In addition, the selection prioritized those strains with distinctive morphological characteristics and differential culture medium requirements.

The Wizard SV Gel and PCR Clean-Up System (Promega Corporation, Madison, WI, USA) kit was used to purify PCR products of strains selected following the manufacturer’s instructions. The purified products were analysed by spectrophotometry on a NanoDrop 2000c equipment.

The strains selected for sequencing were sent to the National Laboratory of Agricultural, Medical, and Environmental Biotechnology (LANBAMA) of the Potosino Institute of Scientific and Technological Research (IPICYT), where Sanger sequencing was performed using the 3500 and 3130 genetic analysers. To ensure reproducibility, each biological sample was sequenced in triplicate. The resulting sequences were assembled using the CAP3 program of UGENE v. 47 [19]. Subsequently, alignment of the ASA.S1 region of the 18S rRNA gene was performed using the ClustalW consensus sequences [20].

Initial phylogenetic analysis employed the Maximum Likelihood method with 1000 bootstrap replicates in MEGA v12.1 software [21], applying the Tamura-Nei (T93) model [22], gamma distribution with invariant sites (G + I) based on Bayesian information criterion (BIC) [23]. Reference sequences used in the genotyping process (Table S1) were sourced from the National Center for Biotechnology Information (NCBI) database [24].

### 2.5. Whole Genome Sequencing and 18S rRNA Mapping of LUDO1

To confirm the phylogenetic classification of a possible new genotype, the entire genome was sequenced, and the 18S rRNA region was mapped to obtain a high-resolution sequence.

DNA was extracted using the commercial kit mentioned in Section 2.3. Whole genome sequencing was conducted with paired-end 150 bp short-reads on an Illumina Novaseq 6000 platform in the LC Sciences Laboratory (Houston, TX, USA). The coverage implemented was 100×. The quality of sequences was assessed using FastQC v0.12.1 [25]. Subsequently, to remove adapters and filter out low-quality reads, Trimmomatic v0.39 was used [26] employing the following parameters: ILLUMINACLIP with Truseq3 adapter sequences, a minimum average quality score of 20 (SLIDINGWINDOW: 4:20), and HEADCROP trim 4 nucleotides from the start of the read. The cleaned reads were assembled *de novo* using the MaSuRCA assembler [27] with an insert size of 150. Assembly quality analysis was conducted using QUAST v5.3.0 [28].

The mapping of the 18S rRNA gene was performed using the sequence of *Acanthamoeba castellanii* Ma ATCC 50370 18S rRNA, sourced from NCBI [24], as a reference with Bowtie2 software v2.5.4 [29]. Finally, the alignment was corrected using Pilon v1.14 [30].

### 2.6. Phylogenetic Reconstruction and Validation

Genotypic classification was determined from two additional phylogenetic analyses based on the 18S rRNA gene sequence. To verify the reliability of the LUDO1 sequence and to rule out false positives, the 18S rRNA sequence obtained from Illumina reads was first incorporated into an alignment of the ASA.S1 region, along with consensus sequences from the isolates in this study and representative sequences of known *Acanthamoeba* genotypes (T1–T23). In addition, an alignment using near-full-length 18S rRNA gene sequences was used to establish the definitive genotypic classification of the LUDO1 strain within the genus *Acanthamoeba* [31]. Phylogenetic trees were constructed using the maximum likelihood method with 1000 bootstrap replicates in MEGA v12.1 software [21]. Analyses were conducted using the T93 and T92 models [22,32], respectively with G + I. Model selection was based on the BIC [23].

## 3. Results

### 3.1. Isolation and Identification of Acanthamoeba Strains

A total of 21 samples were collected during the fall and winter seasons. Eleven samples (52.38%) were positive for *Acanthamoeba* by both morphological and molecular analysis [3]. Only area 5 did not present any positive results (Table 1).

The pH of the samples ranged from 7.2 to 7.8, and the residual Cl concentration ranged from 0.3 to 1.25 mg (Table S2). This characteristic is not associated with *Acanthamoeba* presence or absence in potable water; however, it demonstrates the amoeba’s ability to persist even in bodies of water that meet quality standards suitable for human consumption.

**Figure 2:**
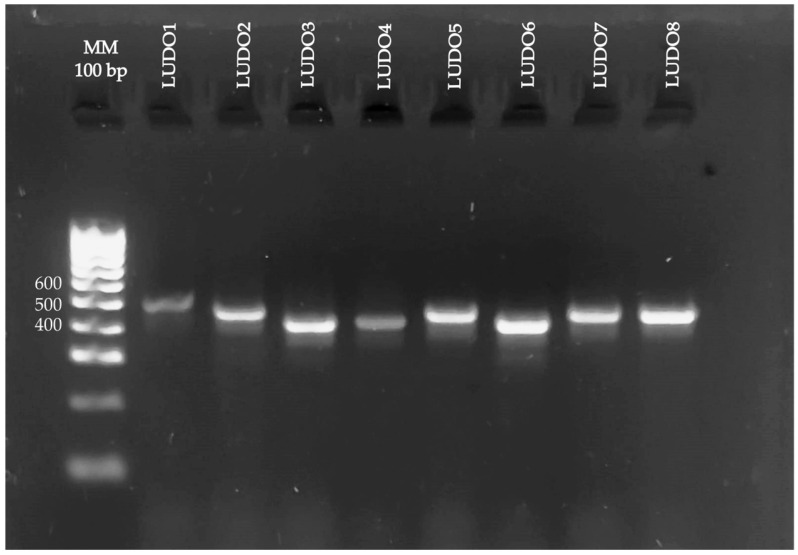
Electrophoresis gel of *Acanthamoeba* positive samples. Lane 1: Hyper-Ladder 100 bp™ molecular weight marker; lanes 2–9: ASA.S1 region amplicons for positive samples.

PCR products showed bands migrating near 500 bp (consistent with the expected range of 423 to 551 bp) (Figure 2), indicating differences in size between strains and suggesting the presence of distinct genotypes. Morphological analysis classified the strains into group II and III; this highlights the potential clinical relevance of the isolates, since these groups encompass the genotypes most frequently recovered in AK and GAE infections.

### 3.2. Variability in Strain Morphology and Growth Requirements

The strategy for selecting strains for ASA.S1 Sanger sequencing prioritized one representative strain per area; however, the evaluation of all positive samples revealed differences in growth and culture requirements among strains. This heterogeneity was critical in the final selection of 8 samples.

The strains from areas 2, 3, 6, and LUDO8 from area seven showed greater adaptability, successfully proliferating in both implemented culture medium (NNE and Cerva’s medium), in contrast to the strains isolated from areas 1, 4, and LUDO7 and LUDO-NS3 strain from area seven, which were unable to maintain viability in Cerva’s medium, revealing stricter requirements, resulting in the selection of two strains for Sanger sequencing of the area 7.

**Figure 3:**
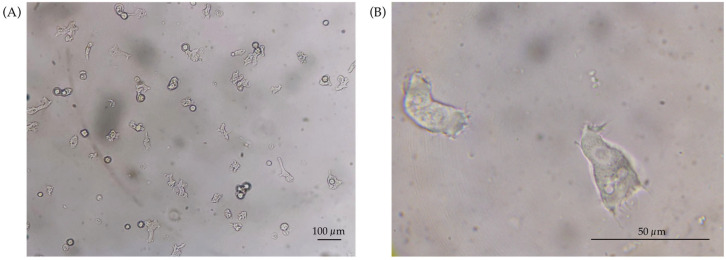
Morphological characterisation of the LUDO4 strain of *Acanthamoeba*. (**A**) General view of the population at 100× magnification, showing a heterogeneous distribution with large trophozoites and emerging cysts. (**B**) Trophozoites visualized at 1000× magnification; the cells shown represent the lower end of the size spectrum (≈33 μm) according to the morphometric analysis of 25 individuals.

Two isolates from area 3 (LUDO3 and LUDO4) were also included due to the notable size difference observed in the trophozoite phase of the LUDO4 strain. The trophozoites of this strain ranged in size from 33 μm to 72 μm (Figure 3). This trait contrasts sharply with that of the other isolates in this study, whose trophozoites showed typical sizes of 15 to 30 μm. 

Taken together, this finding demonstrates that *Acanthamoeba* isolates exhibit biological diversity, even within the same area, supporting the initial idea of the distinct genotypes. In addition, this interesting morphological feature of strain LUDO4 may be related to previously undescribed resistance or mobility traits.

### 3.3. Genotype Distribution

**Figure 4:**
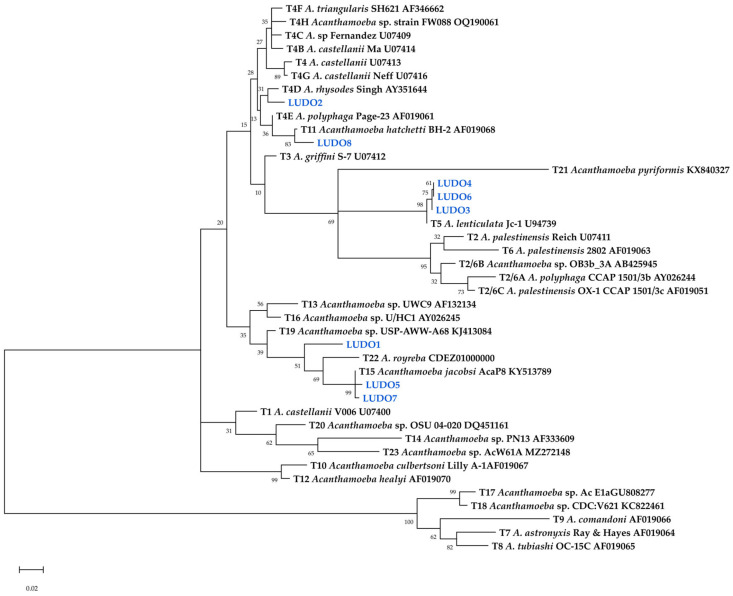
Maximum Likelihood tree for reference sequences of *Acanthamoeba* genotypes from NCBI and ASA.S1 sequences isolated from tap water in Cajeme, Sonora, Mexico. Isolates from this study are highlighted in blue.

**Figure 5:**
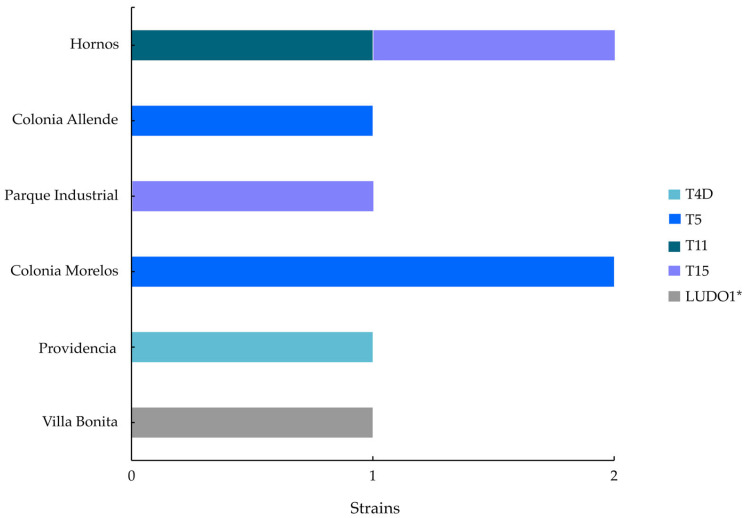
Distribution of the genotypes identified in potable water of Cajeme, Sonora, Mexico, by area of sampling. * Divergent genotype.

The LUDO2-LUDO8 sequences, obtained from the sequencing of the ASA.S1 region of *Acanthamoeba* isolates, were phylogenetically classified into the genotypes T4D, T5, T11, and T15 (Figure 4). Genotype T5 showed the highest frequency and the widest geographic distribution, representing 37.5% of the sequences analysed, followed by T15 with a presence of 25% (Figure 5). These isolates exhibited consistent levels of nucleotide identity. This molecular uniformity contrasts with the notable morphological variation observed in LUDO4, suggesting that the morphological change is not reflected in the primary structure of the 18S rRNA gene.

Furthermore, the LUDO1 sequence formed an independent branch within the monophyletic clade comprising genotypes T15 and T22, supported by a bootstrap value of 51 (Figure 4). In addition, the LUDO1 strain initially showed a sequence dissimilarity greater than 5% compared to its closest relative (Table S3). Therefore, LUDO1 was subjected to a higher-resolution phylogenetic analysis based on the 18S rRNA gene to further investigate its classification as a putative novel genotype.

### 3.4. Genomic Data Characteristics of the Strain LUDO1

The draft genome of strain LUDO1 was obtained using high-quality reads. The assembled genome was estimated at 76.36 Mbp and features strong quality metrics, distributed across 12,899 *contigs*, with no ambiguous bases (“N”). The N50 value reached 21,375, while the L50 was 769. The GC content was measured at 51.77%. Additional statistical details are available in the Supplementary Data (Table S4).

Furthermore, the 18S rRNA region was successfully mapped within the assembled genome. This region was recovered and measured 2353 bp in length.

### 3.5. Identification of a Novel Genotype

The incorporation of genomic data enabled the acquisition of a more robust and unambiguous sequence of the ASA.S1 region from strain LUDO1, measuring 433 bp. This represents an increase of 28 bp compared to the initial Sanger-derived fragment (after removal of primer-derived nucleotides and precise genomic delimitation). The alignment evaluation reinforced the phylogenetic signal of the LUDO1 strain. Phylogenetic reconstruction based on this region, specific to the *Acanthamoeba* genus, revealed a consistent phylogenetic pattern in which strain LUDO1 (Sanger and Illumina data) maintained a distinct taxonomic position (Figure S1). The similarity between the two LUDO1 sequences used in this analysis was 99.5% (Table S5). The additional strains were consistently clustered within genotypes T4, T5, T11, and T15.

Regarding the existing nomenclature, it is worth noting that the divergence between genotypes T17 and T18 observed in this study was below the 5% threshold in both the ASA.S1 fragment and the full-length 18S rRNA gene (Table S6). In the phylogenetic reconstruction, these genotypes formed a clade with high support, characterized by low intergenotypic distance.

**Figure 6:**
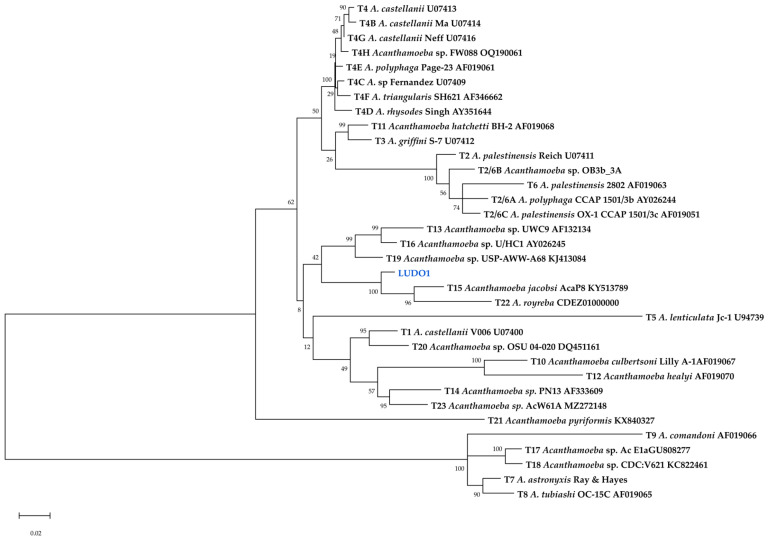
A maximum likelihood phylogenetic tree supporting the identification of a novel genotype T24. Tree based on reference 18S rRNA sequences of *Acanthamoeba* and the complete 18S rRNA region of strain LUDO1, showing the novel genotype positioned as a sister lineage to the T15/T22 clade. Isolates from this study are highlighted in blue.

Finally, phylogenetic analysis based on the full-length 18S rRNA sequence placed the LUDO1 strain as a sister lineage to the T15/T22 clade (Figure 6). Pairwise distance revealed a minimum divergence of 6.77% relative to other genotypes (Table S7). Based on these results, strain LUDO1 is therefore proposed as a novel genotype, designated T24.

**Figure 7:**
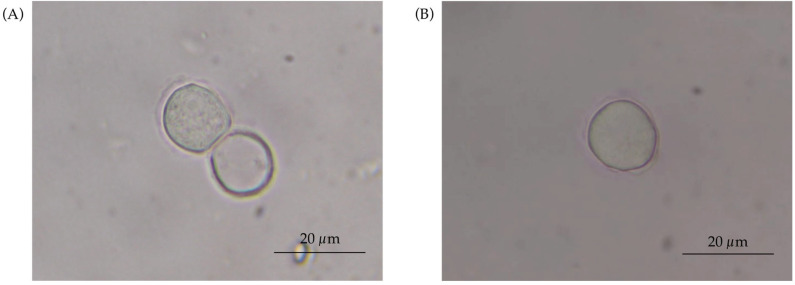
Cyst morphology of *Acanthamoeba* genotype T24 at 1000× magnification. (**A**,**B**) Representative mature cysts showing the characteristic double-walled structure of Group III. (**B**) The ectocyst (outer wall) appears wrinkled or irregular, while the endocyst (inner wall) has a slightly more angular outline than the ectocyst.

Based on morphological characteristics, the novel genotype was assigned to group III. This classification was supported by the rounded shape of the cyst and the presence of a double-layered cyst wall (Figure 7A,B). The endocyst exhibits a slightly more angular outline than the ectocyst. Both layers remained closely apposed. Measurements of 25 cysts revealed an average diameter of 14.04 µm (10–17.5 µm) (Figure 7A,B). The trophozoites displayed variable shapes and multiple acanthopodia, and each cell contained a single nucleus, consistent with typical *Acanthamoeba* morphology. Based on measurements of 25 trophozoites, an average size of 16.52 µm was recorded, with individual measurements ranging from 12.5 to 22.5 µm. Regarding cultivation characteristics, the T24 genotype grew only in NNE medium, with an optimal growth temperature of 37 °C; it could also grow at ambient temperature (~25 °C) and showed encysted growth at 42 °C.

## 4. Discussion

For the first time, identification of *Acanthamoeba* spp. in tap water in Cajeme, Sonora, was carried out, obtaining positive results in 6 of the 7 areas analysed (52.38% of the total samples) (Table 1). The presence of this amoeba raises uncertainty about potable water, which is currently provided to the Cajeme community at a rate of over 1300 L/s to eradicate contaminants and infectious microorganisms [13]. Recent data show that all water potabilization facilities in the municipality of Cajeme use standard clarification and sedimentation [13]. Only Area 5 (Antonio Rosales) had no positive findings, possibly due to its simpler rural distribution network or initial supply conditions, which may limit biofilm and amoeba growth. Studies link the origin of the supply, especially surface or organic-rich water, to higher FLA levels [33,34]. A more thorough analysis of the water sources could provide valuable information for improving water treatment and prevention efforts. Additionally, future studies should consider seasonal variations in the prevalence of the amoeba in the region.

In this region, *Acanthamoeba* and other FLA, such as *Naegleria fowleri* and *Balamuthia mandrillaris*, have been reported from various surface water bodies that also supply these water treatment plants and recreational waters, such as swimming pools, which, by regulation, should not harbour such pathogens [11,15,18]. The incidence of this pathogen in tap water in other regions of the world is becoming increasingly common; in recent years, several studies have reported its presence [2,33,35,36]. In the Metropolitan Area of Mexico City, it has been detected in bottled water at 20% [36] and in domestic water samples at 48.8% [37]. The prevalence values reported in studies, including the present work, are variable; thus, it is important to consider the origin of the samples when relating the presence to tap-water conditions.

Domestic storage systems, such as cisterns and water tanks, have been linked to factors that promote the presence of *Acanthamoeba* [34,38,39]. Sediment accumulation provides nutrients and favourable conditions for the survival of FLAs [38,40]. From a One Health Perspective, *Acanthamoeba* can also act as a vector for other pathogens present in water, such as *Pseudomonas* spp., *Mycobacterium* spp., *Escherichia coli*, and *Listeria monocytogenes* [41,42,43]. Recent evidence also highlights its role in the persistence of emerging fungal threats, such as *Candida haemulonii* complex and *Candida auris*, where the amoebic intracellular environment may facilitate the development of extensively antifungal-resistant phenotypes [44,45]. Although this study did not directly evaluate endosymbionts, the documented capacity of *Acanthamoeba* to protect diverse microorganisms from adverse environmental factors underscores an indirect but significant risk for the transmission of severe infectious diseases within the water supply network [46,47].

Regarding the molecular characterization, several distinct genotypes were identified, as predicted from PCR analyses. Except for the novel genotype reported here, all identified genotypes have been previously associated with severe clinical manifestations. Specifically, genotypes T4 and T5 are linked to potentially lethal GAE [48], while genotypes T4, T5, T11, and T15 are recognised agents of AK [49]. Genotypes T4 and T5 are the most prevalent in the environment, followed by T3, T11, and T15; among these, the T4 genotype is consistently described as the most virulent [1,3,50]. The identification of these diverse genotypes in this study and others suggests that pathogenic *Acanthamoeba* is more common in tap water sources than expected.

A recent study also reported the survival of *Acanthamoeba* in tap water and demonstrated an increased adhesion capacity to host cells. This process is mediated by the coordinated action of the mannose-binding protein (MBP), which acts as a calcium-dependent lectin for initial attachment, and the laminin-binding protein (AhLBP), which provides stable anchoring to the extracellular matrix; thus, the absence of ions in double-distilled water leads to a sharp decline in virulence [51]. According to Corsaro [52], these adhesins have structural variations between genotypes, which could influence binding and recognition efficiency. In this context, the hard water in Cajeme [53] may act not only by favouring the proliferation of this amoeba but also as a selective environmental filter for specific genotypes with optimised MBP activity. This aligns with the exclusive identification of genotypes previously linked to human infections, while simultaneously generating significant uncertainty regarding the pathogenic potential of the novel T24 genotype.

Together, these findings indicate a significant risk of direct and indirect infection for the region’s population, especially among contact lens wearers, as all isolated pathogenic genotypes can cause AK [54].

*Acanthamoeba* strains demonstrated significant variability. Strains LUDO1, LUDO5, and LUDO7, classified as a novel genotype and within genotype T15, respectively, exhibited differential growth in culture medium, failing to proliferate in Cerva medium. This inadaptability is often associated with special specific nutritional or oxygen requirements not met by conventional axenic media, even when dealing with the same species [55,56,57]. While some are known to be “fastidious”, the underlying causes remain unconfirmed and cannot yet be definitively linked to specific genotypes.

Additionally, notable morphological differences were observed in the trophozoites of the LUDO4 strain, with dimensions appearing to be even larger than those typically reported in the literature [3,58]. This morphology was associated with a lower cell division rate, consistent with models where larger trophozoites prioritise biomass accumulation over rapid movement and replication [58]. Such divergence suggests that localised environmental factors in the study area influence these phenotypic characteristics by triggering stress responses, which in turn may involve altered metabolic states that promote cell volume increase [59].

Previous studies, including those on *A. polyphaga,* have linked cellular morphodynamics to the endolysosomal system, where stressors such as nutrient processing or oxidative stress can induce organelle accumulation, increasing cytoplasmic vacuoles and overall cell volume [60,61,62]. Based on this premise, the larger dimensions of LUDO4 could suggest a similar association. However, further analyses are required to confirm these observations, including genomic or transcriptomic comparisons. If confirmed, such approaches would allow us to determine how environmental stress impacts genomic stability [6], leading to a broader understanding of the genus; as demonstrated by the findings of the present study, the 18S rRNA marker ASA.S1, while effective for genotypic identification, does not reflect the phenotypic variability observed within strains.

The integration of genomic data through whole-genome sequencing of the LUDO1 strain provided the necessary resolution to classify this isolate as a novel genotypic variant (T24), exceeding the accepted divergence threshold (>5%) [31]. Using high-precision technologies like Illumina [63] allows for dual objectives: obtaining the 18S rRNA region and a draft of the whole genome in a single sequencing run. While the hypervariable ASA.S1 region remains the cornerstone for genotype identification [17], its short length can complicate the interpretation of deeper evolutionary relationships. Consequently, the full-length 18S rRNA gene was utilised to resolve the phylogenetic placement of LUDO1, consistently positioning it as a sister lineage to the T15/T22 clade [31,64]. These results underscore the relevance of using specific molecular markers employing novel methods, especially when dealing with highly variable genera such as *Acanthamoeba*.

Finally, the observed divergence between T17 and T18 was below the 5% threshold, which is consistent with reports by Corsaro [65], who noted that these genotypes typically form a single lineage in ASA.S1-based trees due to the lack of specific diagnostic signatures in this fragment. While data from both ASA.S1 and the full-length 18S gene show high similarity, the literature agrees that further analyses are required to definitively determine whether they should be consolidated into a single genotype.

## 5. Conclusions

This study provides the first identification of pathogenic species in tap water in Cajeme, Sonora, using a genotypic classification system that proved to be a reliable and effective tool for monitoring strains that could trigger lethal infections in humans. The findings reveal that domestic water storage practices create favourable environments for the proliferation of these amoebae, identifying four previously reported pathogenic genotypes, and a novel genotype, T24, which constitutes a sister lineage to the T15/T22 clade and is morphologically classified in group III. Furthermore, the morphological distinctiveness of strains such as LUDO4 reveals a significant decoupling between morphological plasticity and 18S rRNA molecular uniformity; this suggests that high-resolution genomic data is essential to elucidate the molecular basis of phenotypic plasticity in the genus. These results expand the known biodiversity of the genus and highlight a persistent One Health risk, as these amoebae persist in water meeting official quality standards. Ultimately, this work establishes a critical baseline for epidemiological surveillance and underscores the need for comparative transcriptomics to elucidate the molecular mechanisms of environmental adaptation and pathogenicity in *Acanthamoeba*.

## Figures and Tables

**Table 1:** *Acanthamoeba* positive samples per area.

Area	Household	*Acanthamoeba* Presence	Strain
1	A	✗	NA
	B	✗	NA
	C	✓	LUDO1
2	A	✓	LUDO2
	B	✗	NA
	C	✗	NA
3	A	✗	NA
	B	✓	LUDO3
	C	✓	LUDO4
4	A	✓	LUDO-NS1
	B	✓	LUDO5
	C	✗	NA
5	A	✗	NA
	B	✗	NA
	C	✗	NA
6	A	✗	NA
	B	✓	LUDO-NS2
	C	✓	LUDO6
7	A	✓	LUDO7
	B	✓	LUDO8
	C	✓	LUDO-NS3

NA: not applicable. ✓: positive. ✗: negative.

## Data Availability

Raw FASTQ reads are available in the NCBI Sequence Read Archive (SRA) under BioProject accession PRJNA1392663. The assemblies for ASA.S1 for LUDO1–LUDO8 have been deposited in GenBank under accession numbers PX735250, PX735251, PX735252, PX735260, PX735262, PX735357, PX735358, and PX735360.

## Supplementary Materials

The following supporting information can be downloaded at: https://www.mdpi.com/article/10.3390/tropicalmed11040093/s1, Table S1: Reference sequence used in the genotyping process; Table S2: Sampling areas, pH values, and residual free chlorine concentration of water samples; Table S3: Values of sequence identity between strain LUDO1 with ASA.S1 region by Sanger data and representative genotypes; Table S4: Statistical characteristics of the draft genome of strain LUDO1 obtained using high-quality reads; Figure S1: Maximum likelihood phylogenetic tree supporting the phylogenetic signal of the LUDO1 strain. Table S5: Values of sequence identity between strain LUDO1 with ASA.S1 regions by Sanger and Illumina data and representative genotypes; Table S6: Values of sequence identity between genotypes T17 and T18 with ASA.S1 region and full-length 18S rRNA region; Table S7: Values of sequence identity between strain LUDO1 with 18S rRNA region and representative genotypes.
