# Supplementary material for: Genetic Classification of a Novel Genotype of the Genus Acanthamoeba Isolated from Tap Water in Mexico
Source: Trop Med Infect Dis. 2026 Apr 1;11(4):93. doi: 10.3390/tropicalmed11040093 (PMC13120473; doi:10.3390/tropicalmed11040093)
Supplement: Supplementary file 1 [file tropicalmed-11-00093-s001.zip › tropicalmed-4191885-supplementary.pdf]

Genetic classification of a novel genotype of the genus *Acanthamoeba* isolated from tap water in Mexico

Paloma Camacho-Aguilar [1], Leobardo Daniel Gonzalez-Zuñiga[1], Jose Reyes Gonzalez-Galaviz[2], Fernando Lares Villa[3], Luis Fernando Lares-Jiménez[3], Luis Fernando Lozano Aguirre Beltrán[4], Alejandro Otero-Ruiz[4], Libia Zulema Rodriguez-Anaya[2]*

[1] Departamento de Biotecnología y Ciencias Alimentarias, Instituto Tecnológico de Sonora, Ciudad Obregón 85000, Sonora, México; paloma.camacho207086@potros.itson.edu.mx; leobar-do.gonzalez121194@potros.itson.edu.mx
[2] SECIHTI-Institito Tecnológico de Sonora, Ciudad Obregón 85000, Sonora, México; li-bia.rodriguez@itson.edu.mx; jose.gonzalez@itson.edu.mx
[3] Departamento de Ciencias Agronómica y Veterinarias, Instituto Tecnológico de Sonora, Ciudad Obregón 85000, Sonora, México; flares@itson.edu.mx; luis.lares@itson.edu.mx
[4] Unidad de Análisis Bioinformáticos, Centro de Ciencias Genómicas, Universidad Nacional Autónoma de México, Cuernavaca 62210, Morelos, Mexico; llozano@ccg.unam.mx
[5] Programa de Estancias Posdoctorales por México, Departamento de Ciencias de la Salud, Universidad de Sonora, Ciudad Obregón 85010, Sonora, México; alejandro.otero@unison.mx
* Correspondence: libia.rodriguez@itson.edu.mx; Tel.: 644109000 ext: 2318. 85000.

Supplementary Materials

Table S1. Reference sequence used in the genotyping process.

Reference genotypes	Strain	Accession
T10	*Acanthamoeba culbertsoni* Lilly A-1	AF019067
T11	*Acanthamoeba hatchetti* BH-2	AF019068
T12	*Acanthamoeba healyi*	AF019070
T13	*Acanthamoeba* sp. UWC9	AF132134
T14	*Acanthamoeba* sp. PN13	AF333609
T15	*Acanthamoeba jacobsi* AcaP8	KY513789
T16	*Acanthamoeba* sp. U/HC1	AY026245
T17	*Acanthamoeba* sp. Ac E1a	GU808277
T18	*Acanthamoeba* sp. CDC: V621	KC822461
T19	*Acanthamoeba* sp. USP-AWW-A68	KJ413084
T1	*Acanthamoeba castellanii* V006	U07400
T20	*Acanthamoeba* sp. OSU 04-020	DQ451161
T21	*Acanthamoeba pyriformis*	KX840327
T23	*Acanthamoeba* sp. isolate AcW61A	MZ272148
T2/6A	*Acanthamoeba polyphaga* CCAP 1501/3b	AY026244
T2/6B	*Acanthamoeba* sp. OB3b_3A	AB425945
T2/6C	*Acanthamoeba. palestinensis* OX-1 CCAP 1501/3c	AF019051

T2	*Acanthamoeba palestinensis* Reich	U07411
T3	*Acanthamoeba griffini* S-7	U07412
T4	*Acanthamoeba castellanii*	U07413
T4B	*Acanthamoeba castellanii* Ma	U07414
T4C	*Acanthamoeba* sp. Fernandez	U07409
T4D	*Acanthamoeba rhysodes* Singh	AY351644
T4E	*Acanthamoeba polyphaga* Page-23	AF019061
T4F	*Acanthamoeba triangularis* SH621	AF346662
T4-Neff	*Acanthamoeba castellanii* Neff	U07416
T4H	*Acanthamoeba* sp. strain FW088	OQ190061
T5	*Acanthamoeba lenticulata* Jc-1	U94739
T6	*Acanthamoeba palestinensis* 2802	AF019063
T7	*Acanthamoeba astronyxis* Ray & Hayes ATCC 30137	AF019064
T8	*Acanthamoeba tubiashi* OC-15C	AF019065
T9	*Acanthamoeba comandoni*	AF019066
T22	*Acanthamoeba royreba* ATCC30884	CDEZ01000000

Table S2. Sampling areas, pH values, and residual free chlorine concentration of water samples.

Sampling site	Sample	pH value	Residual Cl concentration
1. Villa Bonita	A	7.2	0.5
	B	7.6	1.5
	C	7.2	0.5
2. Providencia	A	7.8	0.5
	B	7.6	0.5
	C	7.6	0.5
3. Colonia Morelos	A	7.2	1.5
	B	7.2	1.5
	C	7.2	1.5
4. Parque Industrial	A	7.6	0.3
	B	7.6	1.5
	C	7.6	1.5
5. Antonio Rosales	A	7.6	1.5
	B	7.2	1.5
	C	7.6	1.5
6. Colonia Allende	A	7.7	1.5
	B	7.7	1.5
	C	7.6	1.0

7. Hornos	A	7.6	0.5
	B	7.6	0.5
	C	7.6	0.5

Table S3. Values of sequence identity between strain LUDO1 with ASA.S1 region by Sanger data and representative genotypes.

Reference genotypes	LUDO1 ASA.S1 region dissimilarity
T10	0.1797
T11	0.1113
T12	0.2126
T13	0.1337
T14	0.2986
T15	0.0863
T16	0.1251
T17	0.4170
T18	0.4479
T19	0.1305
T1	0.1732
T20	0.1199
T21	0.1328
T23	0.2015
T2/6A	0.2098
T2/6B	0.2211
T2/6C	0.2038
T2	0.2018
T3	0.1455
T4	0.1273
T4B	0.1007
T4C	0.1149
T4D	0.0961
T4E	0.1186
T4F	0.1029
T4G	0.1315
T4H	0.1084
T5	0.1153
T6	0.2103
T7	0.4783

T8	0.3865
T9	0.5106
T22	0.0742

Table S4. Statistical characteristics of the draft genome of strain LUDO1 obtained using high-quality reads.

Statistics without reference	Value
Contigs	12,899 bp
Largest contig	553,899 bp
Total length	78,355,720 bp
N50	21,375
N90	2045
auN	61,138
L50	769
L90	6371
GC%	51.77
N's	0

Figure S1. Maximum likelihood phylogenetic tree supporting the phylogenetic signal of the LUDO1 strain. Constructed using *Acanthamoeba* reference sequences and LUDO1 ASA.S1 regions obtained by Sanger sequencing and mapping of the 18S rRNA region from whole genome sequencing with Illumina reads.

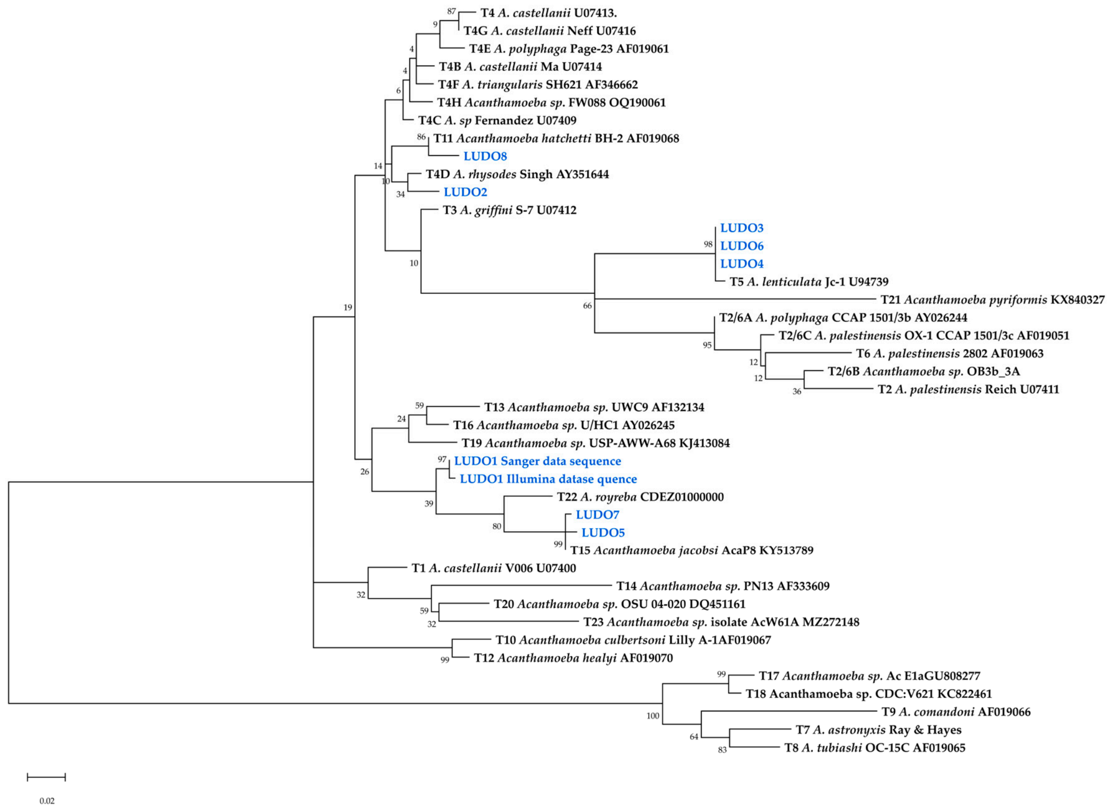

Table S5. Values of sequence identity between strain LUDO1 with ASA.S1 regions by Sanger and Illumina data and representative genotypes.

Reference genotypes	Region dissimilarity of LUDO1	
	ASA.S1 by data Sanger	ASA.S1 by data Illumina
T10	0.1631	0.1606
T11	0.0914	0.0883
T12	0.1983	0.1979
T13	0.1179	0.1169
T14	0.2673	0.2456
T15	0.0817	0.0864
T16	0.1198	0.1187
T17	0.4002	0.3490
T18	0.4275	0.3805
T19	0.1244	0.1271
T1	0.1561	0.1509
T20	0.1059	0.1046

T21	0.1232	0.1169
T23	0.1862	0.1722
T2/6A	0.1995	0.1700
T2/6B	0.2067	0.1766
T2/6C	0.1943	0.1655
T2	0.1911	0.1631
T3	0.1274	0.1245
T4	0.1123	0.1075
T4B	0.0840	0.0814
T4C	0.0989	0.0952
T4D	0.0920	0.0888
T4E	0.0986	0.0949
T4F	0.0889	0.0859
T4G	0.1155	0.1105
T4H	0.0868	0.0840
T5	0.1028	0.0976
T6	0.2058	0.1808
T7	0.4458	0.3962
T8	0.3757	0.3350
T9	0.4845	0.4199
T22	0.0770	0.0890
LUDO1 (ASA.S1 data Sanger)	-	0.0050

Table S6. Values of sequence identity between genotypes T17 and T18 with ASA.S1 region and full-length 18S rRNA region.

	**ASA.S1 region dissimilarity**	**Full-length 18S rRNA region dissimilarity**
	**T18** *Acanthamoeba* sp. CDC: V621	
**T17** *Acanthamoeba* sp. Ac E1a	0.0338	0.0333

Table S7. Values of sequence identity between strain LUDO1 with 18S rRNA region and representative genotypes.

**Reference genotypes**	**LUDO1 18S rDNA region dissimilarity**
T10	0.1728

T11	0.1014
T12	0.2151
T13	0.1264
T14	0.1450
T15	0.0677
T16	0.1112
T17	0.4031
T18	0.3742
T19	0.1089
T1	0.1188
T20	0.1053
T21	0.1690
T23	0.1195
T2/6A	0.1501
T2/6B	0.1399
T2/6C	0.1412
T2	0.1344
T3	0.0967
T4	0.0833
T4B	0.0735
T4C	0.0887
T4D	0.0889
T4E	0.0932
T4F	0.0919
T4G	0.0896
T4H	0.0880
T5	0.2559
T6	0.1573
T7	0.3835
T8	0.3472
T9	0.4852
T22	0.1010
